# Incidence of macrolide–lincosamide–streptogramin B resistance amongst beta-haemolytic streptococci in The Gambia

**DOI:** 10.1186/s13104-017-2427-x

**Published:** 2017-02-23

**Authors:** Ebenezer Foster-Nyarko, Brenda Kwambana, Fatima Ceesay, Kaddijatou Jawneh, Saffiatou Darboe, Sarah N. Mulwa, Buntung Ceesay, Ousman O. Secka, Ifedayo Adetifa, Martin Antonio

**Affiliations:** 1 Vaccines and Immunity Theme, Medical Research Council Unit The Gambia, Banjul, The Gambia; 2 Clinical Microbiology Department, Medical Research Council Unit The Gambia, Banjul, The Gambia; 3 Disease Control and Elimination Theme, Medical Research Council Unit The Gambia, Banjul, The Gambia; 4 Infectious Diseases Epidemiology, London School of Hygiene & Tropical Medicine, London, UK; 5 Faculty of Infectious and Tropical Diseases, London School of Hygiene & Tropical Medicine, London, UK; 6 Microbiology and Infection Unit, Warwick Medical School, University of Warwick, Coventry, UK

**Keywords:** Beta-haemolytic streptococci, Gambia, macrolide–lincosamide resistance

## Abstract

**Background:**

In West Africa, penicillin, macrolide and lincosamide resistance among beta-haemolytic streptococci (BHS) isolates has rarely been described. However, such data are critical to detect and track the emergence of antibiotic resistance.

**Methods:**

Beta-haemolytic streptococci were cultured from clinical specimens from patients attending the clinic at the Medical Research Council Unit The Gambia (n = 217) and kept at −70 °C. Of these, 186 were revived and tested for penicillin susceptibility by disc diffusion and E-test methods, and the D-test for determination of constitutive and inducible macrolide–lincosamide (MLS_B_) resistance phenotypes.

**Results:**

The majority of BHS isolates from infections were group A streptococci (GAS) (126/186, 67.7%). Of these, 16% were from invasive disease (30/186). Other BHS isolated included lancefield groups B (19, 10.2%); C (9/186, 4.8%), D (3/186, 1.6%), F (5/186, 2.7%), G (16/186, 8.6%) and non-typeable (8/186, 4.3%). Prevalence of BHS isolated from blood cultures ranges from 0% (2005) to 0.5% (2010). Most (85, 45.7%) of the isolates were from wound infections. Of the 186 BHS isolates, none was resistant to penicillin and 14 (6.1%) were resistant to erythromycin. Of these, 8 (4.3%) demonstrated constitutive MLS_B_ resistance, and 5 (2.7%) were inducible MLS_B_ resistant. All the inducible MLS_B_ isolates were GAS, and majority of the constitutive MLS_B_ isolates (6/8, 75.0%) were non-GAS.

**Conclusions:**

Beta-haemolytic streptococci, predominantly GAS are associated with a wide range of infections in The Gambia. It is reassuring that macrolide and lincosamide resistance is relatively low. However, monitoring of MLS_B_ resistance is necessary with the global spread of resistant BHS strains.

## Background

The WHO’s global surveillance of antibiotics in 2014 raised concerns that the management of even mild infections in both the community and hospitals is under serious threat as a result of antibiotic resistance [1, 2]. This marks the beginning of what has been described as a post-antibiotic era. Groups A–G Beta haemolytic streptococci (GAS, GBS, GCS, GDS, GFS and GGS) have been reported to be amongst the top 10 causes of invasive bacterial diseases in adults and infants globally [3–5]. Whilst streptococci groups C–G have not previously been considered as important as GBS and GAS, emerging evidence suggests that non-GAS, non-GBS (NABS) invasive disease is increasing [6, 7].

Penicillin is the recommended first line of treatment for streptococcal infections particularly that of GAS and GBS; however macrolides and sometimes lincosamides are recommended as second line options for patients who are allergic to penicillin. Similar to other Gram-positive organisms, macrolide and lincosamide resistance have been reported to be emerging amongst beta- haemolytic streptococcal isolates globally; including reports from Southern Africa [8–12]. This highlights the need to probe resistance patterns to these antibiotic groups.

The occurrence of an erythromycin resistant, clindamycin susceptible isolate indicates the possibility of inducible clindamycin resistance, a phenomenon known as macrolide–lincosamide–streptogramin B (MLS_B_) resistance. For such strains, although clindamycin appears to be susceptible in vitro, treatment failure is likely to occur, and thus it is imperative to screen for Macrolide–lincosamide cross-resistance (D-test) [13]. A positive D-test in such cases indicates cross-resistance to clindamycin. In West Africa, macrolide and lincosamide resistance amongst clinical BHS isolates have rarely been described. However, such data are critical to detect and track the emergence of antibiotic resistance. Therefore, we retrospectively investigated isolates of beta-haemolytic streptococci recovered from patients who were seen at the Medical Research Council Unit The Gambia’s clinic in Fajara between 2004 and 2012. This study reports the phenotypic patterns of macrolide–lincosamide-streptogramin B resistance in these pathogens.

## Methods

### Study area and population

Beta-haemolytic streptococci were isolated from clinical specimens (blood, aspirates, cerebrospinal fluid (CSF), throat swabs, wound swabs, urine and other miscellaneous specimens) collected from patients of all ages who attended the Medical Research Council Unit The Gambia (MRCG) Clinic in Fajara, from 2004 to 2012. Isolates were defined as invasive if they were isolated from blood, CSF or aspirates. The Clinical services department (CSD) of the MRCG has a 42 bed capacity. About 50,000 patients pass through the facilities (ward, out-patient department, gate clinic) every year. The MRCG Clinic facility has been described previously [14, 15].

All beta-haemolytic streptococci were stored at −70 °C. Table 1 summarizes the information on the isolates used in this study.

**Table 1:** Baseline Characteristics of Beta-haemolytic streptococci isolates

	n (%)
Sex	
Female	55 (49.5%)
Male	56 (50.5%)
Total^a^	111 (100%)
Age	
Less than 3 months	5 (5.1%)
3 months to <5 years	32 (32.7%)
5 to <15 years	12 (12.24%)
15 to <35 years	16 (16.3%)
35+ years	15 (15.3%)
Other^b^	18 (18.4%)
Total	98 (100%)
Year isolated	
2004	4 (2.2%)
2005	1 (0.5%)
2006	7 (3.8%)
2007	29 (15.6%)
2008	38 (20.4%)
2009	51 (27.4%)
2010	24 (12.9%)
2011	21 (11.3%)
2012	11 (5.9%)
Total	186 (100%)
Source of isolation	
Blood culture	21 (11.3%)
Ear swab	34 (18.3%)
Aspirates	8 (4.3%)
Throat swab	15 (8.1%)
Wound swab	85 (45.7%)
CSF	1 (0.5%)
Urine	9 (4.8%)
Others^c^	13 (7.0%)
Total	186 (100%)

^a^75 isolates, although tested for antibiotic susceptibility, had missing records for sex and were thus excluded from the table

^b^Other comprised 18 subjects, for whom age was recorded in source document as child (3/18) or adult (15/18)

^c^Others comprised high vaginal swabs, nasal swabs, oral swabs, serum, skin swabs, sputum, eye swabs

The Joint Gambia Government and MRCG Ethics Committee approved the study.

### Microbiological methods

The isolation of BHS from clinical specimen was carried out on 5% sheep Blood agar plates supplemented with gentamicin using standard microbiology techniques as described previously [16]. Isolates were retrieved, plated out on 5% sheep blood agar plates and confirmed by using the Streptex^®^ Streptococcal grouping kit (Remel & Oxoid, Thermo Fisher Scientific, Basingstoke, UK) as Lancefield groups A, B, C, D, F and G.

All isolates were tested for their susceptibility to Penicillin discs (10 units) (BD Oxoid, Basingstoke, United Kingdom) using the Kirby-Bauer method. Mueller–Hinton agar supplemented with 5% sheep blood (MHA) was used for the susceptibility testing. All isolates showing resistance or intermediate sensitivities to penicillin were subjected to the E-test. All the BHS isolates were further screened by the D-test method using 15 µg Erythromycin and 2 µg Clindamycin discs to determine macrolide–lincosamide-streptogramin resistance following Clinical and Laboratory Standards Institute (CLSI) guidelines [13].

All interpretation of antibiotic susceptibilities were done using CLSI breakpoints [13]. All reagents and the disc dispenser were obtained from BD Oxoid, Basingstoke, UK, except for E-test strips which were obtained from Biomerieux, UK Limited. The MRCG submits to the external quality assurance programme of the UK National External Quality Assessment Service [17] and is a World Health Organization (WHO) Regional Reference Laboratory for invasive bacterial diseases (IBD).

### Data management

Patient information and BHS culture data were collected from MRCG clinic’s microbiology records. The total number of blood and CSF cultures performed per year over the study period was similarly collected from the clinical microbiology records. From the clinic data, age was recorded as a continuous variable but was conveniently categorized into 5 groups. All these variables were summarized using frequency counts and percentages, n (%). Associations between baseline variables age and gender as well as responses to the 3 antibiotics with group were explored using Fishers exact tests.

## Results

This study retrospectively analyzed clinical isolates of BHS derived from patients who attended the MRCG’s clinic from 2004 to 2012. Of 217 isolates collected over the study period, 186 (85.7%) were successfully revived for further testing. Only one isolate per patient was included in the study.

Table 1 summarizes the characteristics of the patients from whom BHS were isolated. Laboratory records clinical isolates were mostly sparse for 2004 and 2005; and for some years not all clinical information was available. Of the patients who had complete clinical records, there were approximately equal numbers of males and females. Most (32, 26.1%) of the samples came from 3 months to 5 year olds; and from wound infections (32, 26.1%). The overall distribution of lancefield groups A-G is as follows: Group A dominated (126, 67.8%); followed by GBS (19, 10.2%); GGS (16, 8.6%); GCS (9, 4.8%); NT (8, 4.3%) GFS (5, 2.7%) and GDS (3, 1.6%). Approximately 16% of these isolates were from invasive disease (30/186) i.e. aspirates, blood cultures and CSF (Table 1). These comprised GAS (19/30, 63.3%); NT (3/30; 10%); GBS (2/30; 6.7%); GCS (2/30; 6.7%); GDS; 2/30; 6.7%) and GFS (2/30; 6.7%). The majority of BHS isolates were from wound infections.

Of the major BHS groups (GAS, GBS and GGS), the proportions of isolates amongst males and females differed significantly (p = 0.03), with GAS having the highest proportion (84/100; 84%) (Table 3).

Of 30 isolates recovered from sterile sites, 9 (30%) were of BHS groups C-G; namely 2 GFS, 2 GDS, 2 GCS and 3 were non-typeable BHS.

Of the 186 BHS isolates, 13 (6.9%) across all lancefield groups were resistant to penicillin by the disc diffusion method; however E-test resolved all of these as sensitive (100%). Overall, 14 out of the 186 isolates (6.1%) were resistant to erythromycin. The phenotypic profiles of macrolide–lincosamide resistance of the erythromycin resistant isolates are presented in Table 2. Notably, all isolates that were iMLS_B_ isolates belonged to lancefield group A. However, all 3 isolates that exhibited clindamycin resistance only were non-GAS (1 GDS, 1GBS and 1 GCS). Similarly, as can be seen from Table 2, 6 out of 8 (75%) of the cMLS_B_ isolates that were found in this study were non-GAS (1 GDS, 3GDS, and 2 GBS).

**Table 2:** Phenotypic resistance patterns amongst clinical BHS isolates (n = 186)

	n (%)
Inducible MLS_B_	5 (2.7%)
Constitutive MLS_B_	8 (4.3%)
Macrolide resistance only	2 (1.1%)
Clindamycin resistance only	3 (1.6%)
Macrolide–lincosamide susceptible	168 (90.3%)
Total	186 (100%)

*MLS*_*B*_ Macrolide–lincosamide–streptogramin B resistance phenotype

The resistance of isolates to erythromycin did not appear to differ across BHS groups (p = 0.4) (Table 3). That of clindamycin showed some borderline difference between the BHS groups (p = 0.04); with the greater proportion of GAS being sensitive to this antibiotic. Penicillin resistance however did not differ amongst GAS, GBS and GGS (p = 0.3).

**Table 3:** Epidemiology and antibiotic resistance rates amongst the predominant BHS groups

Characteristic	Category	GAS	GBS	GGS	Total	p value
Sex, n (%)	Female	47 (88.7)	2 (3.8)	4 (7.5)	53 (100.0)	0.03
	Male	37 (78.7)	9 (19.2)	1 (2.1)	47 (100.0)	
	Total	84 (84.0)	11 (11.0)	5 (5.0)	100 (100.0)	
Age, n (%)	≤3 month	4 (80.0)	1 (20.0)	0 (0.00)	5 (100.0)	0.03
	3 month to ≤5 years	26 (100)	0 (0.0)	0 (0.0)	26 (100.0)	
	5 to ≤15 years	12 (100.0)	0 (0.0)	0 (0.0)	12 (100.0)	
	15–35 years	11 (68.8)	3 (18.8)	2 (12.5)	16 (100.0)	
	>35 years	9 (69.2)	3 (23.08)	1 (7.69)	13 (100.0)	
	Others^a^	12 (70.6)	3 (17.7)	2 (11.8)	17 (100.0)	
	Total	74 (83.2)	10 (11.2)	5 (5.6)	89 (100.0)	
Penicillin resistance, n (%)	Resistant	5 (71.4)	2 (28.6)	0 (0.0)	7 (100.0)	0.3
	Sensitive	121 (78.57)	17 (11.0)	16 (10.4)	154 (100.0)	
	Total	126 (78.3)	19 (11.8)	16 (9.9)	161 (100.0)	
Clindamycin resistance, n (%)	Intermediate	1 (100.0)	0 (0.0)	0 (0.0)	1 (100.0)	0.04
	Resistant	2 (33.3)	3 (50.0)	1 (16.7)	6 (100.0)	
	Sensitive	123 (79.9)	16 (10.4)	15 (9.7)	154 (100.0)	
	Total	126 (78.3)	19 (11.8)	16 (9.9)	161 (100.0)	
Erythromycin resistance, n (%)	Intermediate	6 (66.7)	2 (22.2)	1 (1.1)	9 (100.0)	0.4
	Resistance	8 (66.7)	2 (16.7)	2 (16.7	12 (100.0)	
	Sensitive	112 (80.0)	15 (10.7)	13 (9.3)	140 (100.0)	
	Total	126 (78.3)	19 (11.8)	16 (9.9)	161 (100.0)	

^a^Other comprised 18 subjects, for whom age was recorded in source document as child (3/18) or adult (15/18)

## Discussion

Here we have given the first report of erythromycin resistance and macrolide–lincosamide–streptogramin cross-resistance in beta-haemolytic streptococci from West Africa. We find an overall erythromycin resistance of 6.1% amongst clinical isolates in The Gambia, and no penicillin resistance amongst beta-haemolytic streptococci isolates in our study population.

These findings are similar to what has been reported from other middle to low income settings. In a Turkish study, 9% of clinical GAS isolates were found to be resistant to erythromycin; and all of them were inducibly resistant to clindamycin [10]. In India, 12% of groups C and G isolates from carriage and clinical sources combined were reported to be erythromycin resistant [11].

Contrary to what we found, rates of erythromycin resistance of up to 44% have been reported amongst clinical GBS isolates from Taiwan [8]. A Korean study of macrolide resistance amongst BHS isolates from a tertiary hospital also reported a high rate of 37% resistance of GBS to erythromycin, but a reduced rate of 5% resistance of GAS to erythromycin, and no clindamycin resistance [18]. In that study, GCS and GGS isolates recorded erythromycin resistance of 9.4 and 3.1% respectively.

Comparison of these and published figures suggest that erythromycin and clindamycin resistance varies greatly between different geographical areas; notably in line with differing selective pressure from antibiotic use, along with other factors [19, 20]. Widespread use of antibiotics and intrapartum prophylaxis are contributing to the emergence of macrolide resistance [19, 21]. Antibiotic use drives the selection pressure that results in the emergence of resistance; however this has been linked with high consumptions beyond a critical theoretic threshold [19, 20]. In The Gambia, similar to other West African countries, intrapartum prophylaxis is not practiced. Clindamycin use is restricted, although since 2013 erythromycin is routinely used to treat infections of the skin and lower respiratory tract [22]. Resistance amongst BHS to clindamycin and erythromycin has not been reported prior to this study. Moreover, access to antibiotics in the Gambia is relatively limited due to reasons of cost. These may account for the low level of clindamycin and erythromycin resistance that we found amongst our isolates in this study. The annual patient population at MRCG clinic is about 2.5% of the Gambian population (~1.9 million).

Similar to our findings, no resistance to erythromycin and clarithromycin were found amongst 64 Gambian *H. pylori strains* isolated from gastric biopsies [22]. We previously reported the prevalence of erythromycin resistance amongst 162 carriage isolates of GBS to be 3.1% [23]. In another study involving 151 *Staphylococcus aureus* isolates, no resistance to erythromycin was found, and the highest resistance rate was 34% of overall isolates; to tetracycline [24]. Similarly high levels of susceptibilities to a wide range of antibiotics have been reported amongst invasive strains of *Streptococcus pneumoniae* and *Salmonella* from the Gambia [14, 25].

Of note, all the iMLS_B_ isolates that were found in this study were GAS. However, all 3 isolates that exhibited clindamycin resistance only were non-GAS (1 GDS, 1GBS and 1 GCS). Similarly, 6 out of 8 (75%) of the cMLS_B_ isolates that were found in this study were non-GAS (1 GDS, 3GDS, and 2 GBS). Although erythromycin resistance across the three dominant BHS groups (GAS, GBS and GGS) did not differ significantly, we observed some difference between the sensitivity of clindamycin across the BHS groups. Our findings suggest that BHS antibiotic resistance, particularly due to NABS may be under-reported in West Africa.

The prevalence of BHS isolated from blood ranged from 0% (2005) to 0.5% (2010). Of 30 isolates recovered from sterile site, 9 (30%) were of BHS groups C-G; namely 2 GFS, 2 GDS, 2 GCS and 3 were non-typeable BHS. Although the proportions found were quite small, our study shows that this group of bacteria may constitute neglected disease. In the US, an active population and laboratory-based surveillance found 489 invasive NABS from hospitalized patients over a 2 year period [6]. Mortality rates of 3–10% due to NABS have been reported from Australia [7].

There are several limitations to the design and laboratory methods used in this study that limit its application to similar settings. First, the clinical isolates were chosen from patients who visited the MRCG clinic at Fajara during the study period; thus the prevalence of macrolide resistance we found may not be representative of the entire Gambian population. Furthermore, for some clinical isolates, some clinical information were missing from the laboratory records. Despite these limitations, this study provides important baseline information on the patterns of macrolide and lincosamide resistance amongst clinical isolates of BHS in the Gambia. Our findings indicate that monitoring of macrolide–lincosamide cross-resistance is warranted; and support the routine screening of in clinical isolates before treatment. It would be desirable for future studies to investigate the genotypes of resistance, and correlate these with the resistance that has been reported here.

## Conclusions

Beta-haemolytic streptococci, predominantly GAS are associated with a wide range of infections in The Gambia. It is reassuring that macrolide and lincosamide resistance is relatively low. However, monitoring of MLS_B_ resistance is necessary with the global spread of resistant BHS strains.

## Abbreviations

BHS: beta-haemolytic streptococci
CSF: cerebrospinal fluid
CLSI: Clinical Laboratory Standards Institute
GAS: group A streptococci
GBS: group B streptococci
GCS: group C streptococci
GDS: group D streptococci
GFS: group F streptococci
GGS: group G streptococci
MLS_B_: Macrolide–lincosamide–streptogrammin B
MRCG: Medical Research Council Unit The Gambia
NABS: non-GAS, non-GBS streptococci
WHO: World Health Organisation
